# Challenges to measuring and achieving shared decision‐making in practice

**DOI:** 10.1111/hex.12659

**Published:** 2018-01-03

**Authors:** Sarah T. Hawley

**Affiliations:** ^1^ University of Michigan and Ann Arbor VA Health System

Shared decision‐making (SDM) has been promoted for improving patient‐centered care, specifically ensuring that decisions are made after careful consideration of the pros and cons of options, patients’ underlying values and preferences, and patients are actively involved in shared conversations with their clinicians.^1^, ^2^, ^3^ Despite international support for the concept of SDM, challenges to achieving it have been noted. Some include: (i) measuring and operationalizing the concept of “shared decision‐making,” (ii) identifying tools to help support SDM, (iii) how to best integrate SDM into the clinical encounter.

Several articles from this issue of Health Expectations highlight some of these challenges. Two manuscripts touch on the measurement of SDM, both reporting on the use of established measures in the field.^4^, ^5^ Menear and colleagues evaluated the use of the OPTION scale in primary care settings. Two key factors were reported to be associated with higher OPTION scores, indicative of more SDM: (i) patient report of decision conflict, and (ii) longer duration of visits. While encouraging that more SDM was seen in consultations where patients reported more conflict, the longer length of the visits underscores the challenges of practicing SDM in a busy setting.

Forcino and colleagues also reported on the use of a validated tool for assessing patient report of SDM (i.e., the CollaboRATE) in three primary care settings. They found clinical workflow issues reduced its effective implementation. The authors conclude that assessing patient experiences through a survey such as CollaboRATE could become burdensome, and recommend episodic use of short measurement tools. These two papers raise questions regarding how to best integrate more SDM around complex problems while also effectively measuring it in the context of actual clinical practice, in time‐limited consultations, particularly in primary care where many problems may be presented for discussion.

Leppin and colleagues’ paper reports that patient involvement in decision‐making varied depending on the type of decision being made. Moreover, in many cases where decision control could not be delegated to one person (either patient or clinician), such as decisions about self‐management, decisions were left unmade. The authors conclude by suggesting that SDM may be better achieved by a shift in the content of conversations to focus on explicit decisions about things that may not always appear to require “decisions.”

Measuring SDM more often, as suggested by Forcino et al., may improve the ability to integrate into the clinical workflow. This could also slow things down to focus on assessing all issues, not just those that appear to require a specific decision, as suggested by Leppin et al. However, Menear et al.'s work suggests this approach could miss those visits where more SDM occurs, specifically those with more conflict that take longer. These three papers highlight the challenge of operationalizing and measuring SDM in practice.

Another challenges relates to the clinical implementation of tools to support SDM. Two studies in this issue focus on the use decision aids (DAs) to promote SDM. Holmes‐Rover and colleagues report on the use of a DA for treatment for localized prostate cancer. The authors conducted qualitative analysis of audio recordings and found that, rather supporting SDM, the booklet appeared to support patients’ asking of narrow and specific medical questions. They suggest that perhaps a role of a DA is to clarify issues for patients in advance, thus reducing the need for “shared decision‐making” across the entire encounter. In some ways, this finding reflects that of Menear and colleagues; specifically that those issues about which patients are most conflicted are those that receive the most attention, rather than the overall decision being made.

Wildeboer and colleagues explored the views of general medicine practice staff regarding use of a DA to support SDM for people with type 2 diabetes. The authors noted that despite the general staff support for the DA, its actual use in clinical practice was limited. Through qualitative interviews, they identified positive aspects of the DA, in that it helped staff identify and change their paternalistic approaches. Yet, they also found that DA use was low when staff reported conflict with the content of the DA. This finding underscores the importance of including the potential beneficiaries of the tool in the process to ensure the resultant tool will be acceptable and useful in clinical practice.

Two other papers in this issue highlight the importance of inclusion of relevant parties in the decision‐making process. Lamahewa and colleagues conducted a qualitative study assessing the challenges to decision‐making at the end of life for people with dementia, among both practitioners and caregivers. They concluded that there is a need to clarify the roles of all involved earlier in the process to support SDM at the end of life. Lipson‐Smith and colleagues identified an important and understudied aspect of SDM, specifically when one of the key players (the patient and/or family members) are not native English speakers. The authors note the importance of addressing these populations.

This issue of HEX includes many other papers that address the importance of advocacy, diversity and public involvement across a range of conditions and issues, which to some extent are all important aspects of SDM. The papers described in this briefing are those that specifically raise awareness of some of the challenges to measuring SDM, and to actually achieving it in clinical practice. While tools such as patient education and formal decision aids will continue to be needed, manuscripts in this issue highlight that delivering these tools and measuring their impact across diverse populations will remain important areas for continued research.
